# A theory-driven rationale for EMDR therapy among patients with a personality disorder

**DOI:** 10.3389/fpsyg.2026.1905539

**Published:** 2026-08-12

**Authors:** Christina W. Slotema, Ad de Jongh

**Affiliations:** 1 Department of Clinical psychology, Erasmus University Rotterdam, Rotterdam, Netherlands; 2 Department of Personality disorders, Parnassia Psychiatric Institute, The Hague, Netherlands; 3 Research Department, PSYTREC, Bilthoven, Netherlands; 4 Academic Centre for Dentistry Amsterdam (ACTA), University of Amsterdam and VU University Amsterdam, Amsterdam, Netherlands; 5 School of Health Sciences, University of Salford, Manchester, United Kingdom; 6 Institute of Health and Society, University of Worcester, Worcester, United Kingdom; 7 School of Psychology, Queen’s University, Belfast, United Kingdom; 8 Department of Nursing, Midwifery & Health, Northumbria University, Newcastle, United Kingdom

**Keywords:** adaptive information processing model, eye movement desensitization and reprocessing, personality disorder, remission, trauma-focused therapy

## Abstract

Personality disorders (PDs) are prevalent and debilitating conditions characterised by enduring disturbances in self-functioning, affect regulation, impulse control, and interpersonal relationships. Although several evidence-based psychotherapies are available, their accessibility and efficiency remain limited due to long treatment durations, high intensity, substantial dropout rates, and restricted treatment capacity. The purpose of the present paper is to present an alternative developmental and treatment perspective on PDs and to review the available literature on the effects of eye movement desensitization and reprocessing (EMDR) therapy in this population. This approach is grounded in evidence indicating that adverse childhood experiences, including emotional abuse, neglect, and other forms of early adversity, are highly prevalent among individuals with a PD and are strongly associated with the development and persistence of personality pathology. Shapiro’s Adaptive Information Processing model provides a theoretical framework for understanding this association. In line with this trauma-focused perspective, EMDR therapy is increasingly being studied as a treatment for PDs. The available research on the treatment of PDs indicates that EMDR therapy is associated with significant reductions in posttraumatic stress symptoms, alongside meaningful improvements in PD symptomatology and overall personality functioning. Remission rates for PDs were substantial in several studies, including those of patients without comorbid posttraumatic stress disorder (PTSD). In addition, EMDR therapy was found to be safe, with low dropout rates in most studies and no evidence of increased self-harm, suicidality, or hospitalization. Accordingly, the existing findings support the hypothesis that trauma-related pathogenic memories play a central role in PD pathology and suggest that EMDR therapy may be an effective, efficient, and accessible treatment option for individuals with a PD. Furthermore, large-scale comparative studies are needed to clarify its role in standard PD care.

## Introduction

Personality disorders (PDs) are characterised by enduring disturbances in self-and interpersonal functioning, including maladaptive patterns in the perception and interpretation of oneself, other people, and events, as well as dysregulation in affect, impulse control, and interpersonal responses (American Psychiatric Association, 2013). PDs are highly prevalent, with estimates ranging from approximately 12% in the general population (Volkert et al., 2018) to nearly 50% in mental health care settings (Beckwith et al., 2014). Their impact on daily functioning is substantial, affecting work, education, friendships, and intimate relationships (Skodol, 2018). The resulting burden is considerable, not only for the individuals themselves but also for their families and broader social environments.

Psychotherapy is generally regarded as the first-choice treatment for PDs. Over the past few decades, several evidence-based psychotherapeutic interventions have been developed, particularly for borderline personality disorder (BPD; De Jongh and Slotema, 2026). These approaches are largely grounded in the assumption that PDs arise from enduring maladaptive personality traits and deficits in emotion regulation, and they aim to target these core features through different therapeutic mechanisms (Leichsenring et al., 2024; Stoffers-Winterling et al., 2022). Currently, a range of psychotherapeutic models have demonstrated efficacy, although no single treatment has consistently proven to be superior to the others (Setkowski et al., 2023). For example, psychodynamic psychotherapy focuses on exploring and working through unconscious conflicts and relational patterns within the therapeutic relationship (Gabbard, 2004). Mentalization-Based Therapy aims to enhance the capacity to understand one’s own and others’ mental states, thereby improving affect regulation (Bateman and Fonagy, 2016). Schema Therapy emphasizes the identification and modification of maladaptive schemas and entrenched behavioral patterns (Young et al., 2003), while Dialectical Behavior Therapy (DBT) teaches skills to manage intense emotions, improve interpersonal functioning, and tolerate distress through a balance of acceptance and change (Linehan, 1987).

Despite their demonstrated efficacy and major contributions to the field, these interventions have limitations (Stoffers-Winterling et al., 2022). The treatment duration is typically long, often at least 1 year, and the intensity is high, frequently involving at least two sessions per week (Leichsenring et al., 2024). Consequently, these therapies place substantial demands on mental health care systems and contribute to long waiting times. In routine clinical practice, fewer than 25% of patients with (borderline) PD receive psychotherapy as a first-line intervention (Hermens et al., 2011). Barriers include the limited availability of trained therapists, difficulties in organising care in accordance with guideline recommendations, poor treatment attendance, and the need to stabilise and motivate some patients before psychotherapy can begin (Cloitre et al., 2011; Harned et al., 2014). In addition, dropout rates remain high, with approximately 30% of outpatients prematurely discontinuing treatment (Iliakis et al., 2021). A substantial proportion of patients do not derive sufficient benefit from psychotherapy (Cuijpers et al., 2024; Leichsenring et al., 2024; Stoffers-Winterling et al., 2022). Furthermore, most research has focused on BPD, although there is a growing body of literature addressing cluster C PDs (e.g., Baljé et al., 2024). Taken together, these limitations underscore the urgent need for more efficient, accessible, and scalable interventions for individuals with PDs to address these issues.

A promising alternative is a trauma-focused approach to the treatment of PD. The rationale for this approach lies in the significant role that traumatic and stressful memories appear to play in the onset and persistence of PD symptoms, particularly when these memories continue to interfere with daily functioning. Accordingly, since the late 2010s, eye movement desensitisation and reprocessing (EMDR) therapy has been increasingly investigated in patients with PDs (De Jongh et al., 2024a). Because an overview of scientific research in this field is lacking, the purpose of the present paper is to present a developmental model of PDs and, in line with this perspective, provide an overview of the available literature on the effects of EMDR therapy in this population.

### Personality disorders and adverse childhood experiences

A substantial body of evidence indicates that the onset, maintenance, and treatment of PDs are fundamentally rooted in adverse childhood experiences (ACEs), defined as potentially traumatic events occurring before the age of 18, including sexual, physical, and emotional abuse, physical and emotional neglect, and household dysfunction, such as substance misuse, domestic violence, or parental separation (Felitti et al., 1998; De Jongh et al., 2024a). Such experiences may result in “toxic stress,” which can disrupt brain development and increase the risk of long-term physical, mental, and behavioral health issues. The seminal ACE study by Felitti et al. (1998), based on a large survey of health maintenance organisation members, demonstrated the widespread prevalence and long-term consequences of childhood adversity. More than two-thirds of the participants reported at least one ACE, and one in five reported three or more ACEs. Importantly, this study revealed a graded dose–response relationship between the number of ACEs and the severity of physical and mental health problems in adulthood. In particular, patients with BPD are known to report high rates of ACEs. For example, a meta-analysis of case–control studies found that individuals with BPD were nearly 14 times more likely to report childhood adversity than non-clinical controls (Porter et al., 2020). More than 70% of participants with BPD reported at least one ACE. Physical neglect was the most frequently reported type (48.9%), followed by emotional abuse (42.5%), physical abuse (36.4%), sexual abuse (32.1%), and emotional neglect (25.3%). High rates of childhood adversity are not limited to BPD but extend across PDs more broadly (Broekhof et al., 2024; Kühner et al., 2025). Studies have reported neglect in 85% and abuse in 73% of patients with PDs (Battle et al., 2004; Johnson et al., 1999).

### Neurodevelopmental consequences of adverse childhood experiences

When ACEs occur during critical developmental periods, they may interfere with the maturation of brain regions involved in cognition, affect regulation, threat detection, and self-referential processing (Cattane et al., 2017; Herzog et al., 2022; McCrory and Viding, 2015; Teicher and Samson, 2016). The extent of these disruptions depends on the severity, duration, and timing of the adversity (Hughes et al., 2017; Putnam et al., 2013; Weber et al., 2008; Zarse et al., 2019; Schalinski et al., 2016). These neurodevelopmental changes are closely associated with the emergence of negative beliefs about oneself and others, often characterised by persistent expectations of rejection, danger, and unworthiness (Schalinski et al., 2016; McCrory et al., 2022). Exposure to multiple forms of trauma, including neglect, is associated with greater symptom severity, particularly in affect regulation and interpersonal functioning, which constitute the core psychopathology of PDs (Hughes et al., 2017).

### The adaptive information processing model

The adaptive information processing (AIP) model, developed by Shapiro (2001), offers a theoretical framework for understanding the persistence of psychological symptoms and interpersonal dysfunctions. According to this model, the brain normally processes and integrates new experiences into adaptive memory networks, resulting in memories that no longer elicit intense distress. However, when an event is highly stressful or traumatic, this processing may be disrupted, leaving the experience stored in a maladaptive form. In such cases, memory retains the affective, somatic, and cognitive elements that were present at the time of the original experience (Shapiro, 2001; De Jongh et al., 2024b). When current events, often interpersonal in nature, activate these maladaptively stored memories, intense emotional reactions, physiological arousal, and negative cognitions about the self and others can easily be triggered (De Jongh and Slotema, 2026). Depending on the severity and duration of ACEs, such activation may occur more frequently and intensely. This persistent sensitivity to stress has been conceptualised as *latent vulnerability*, resulting in an enduring alteration in emotional and interpersonal functioning that continues to shape responses later in life (McCrory and Viding, 2015).

### The influence of pathogenic memories on personality functioning

Chronic exposure to ACEs may lead to the formation of maladaptive memory networks that increasingly shape perceptions, affect, and behavior. When activated by present-day events, these networks elicit responses rooted in past experiences rather than the current situation (De Jongh et al., 2024a; De Jongh and Slotema, 2026). In particular, activation of attachment-related pathogenic memory networks may evoke intense affective states and negative self-referential beliefs, such as expectations of being unwanted, rejected, or abandoned (De Jongh et al., 2024a). From this perspective, maladaptive behaviors may emerge in response to such activation, including anger outbursts, impulsive risk-taking, frantic efforts to prevent abandonment, rejection of others, or interpersonal withdrawal. These behaviors may, in turn, provoke negative reactions from significant others, such as criticism, emotional distance or anger attacks. Such responses can, in turn, reactivate the same maladaptive memory networks, thereby confirming and consolidating dysfunctional beliefs (De Jongh et al., 2024a). In this way, current interpersonal stressors not only trigger emotional dysregulation and relational problems but may also become new adverse experiences incorporated into existing maladaptive networks. Over time, this recursive process may intensify and stabilise symptoms such as low self-esteem, emotional instability, impaired emotion regulation, and persistent interpersonal dysfunction, ultimately resulting in enduring patterns that meet the diagnostic criteria for a PD. Thus, the AIP model provides a plausible explanatory framework for the central role of ACEs in the development and maintenance of PD symptoms (De Jongh et al., 2024a).

### EMDR therapy as an etiological intervention

In line with this trauma-focused perspective, trauma-focused therapies have increasingly been studied in patients with a PD (De Jongh and Slotema, 2026). This applies to EMDR therapy in particular. EMDR is a structured treatment designed to reduce the distress associated with traumatic memories. During treatment, the patient focuses on emotionally disturbing memories while simultaneously engaging in working memory taxing tasks, most commonly through guided eye movements (De Jongh et al., 2024b). This process has been found to facilitate memory reprocessing, thereby reducing emotional intensity, resulting in a decrease in related complaints (Alting van Geusau et al., 2025). The AIP model, which forms the theoretical foundation of EMDR therapy, posits that the brain has an inherent capacity to process experiences toward adaptive resolution, but that traumatic or highly stressful experiences may become dysfunctionally stored (Shapiro, 2001). According to the AIP model, through EMDR therapy, these maladaptively stored memories can be reprocessed and integrated into more functional and less distressing memory networks.

Meta-analytic evidence indicates that EMDR therapy is effective not only for acute and chronic PTSD following traumatic experiences in adulthood (De Jongh et al., 2024b; Yunitri et al., 2023) but also for memories related to early childhood trauma (Ajele and Idemudia, 2025). Therefore, major international guidelines, including those of the National Institute for Health and Care Excellence (2018), the International Society for Traumatic Stress Studies (2018), and the U.S. Department of Veterans Affairs and the Department of Defense (2023) have recommended EMDR therapy as a first-line treatment for trauma-related mental health conditions, most specifically post-traumatic stress disorder (PTSD). In 2019, the first study on EMDR therapy in patients with PDs and comorbid PTSD was published (Slotema et al., 2019). Since then, research in this field has expanded considerably.

### Effects of EMDR therapy in patients with a personality disorder

To date, nine studies have examined the effects of EMDR therapy in this population, including four randomised controlled trials (RCTs), four uncontrolled trials, and one non-concurrent multiple-baseline study (see Table 1). Five studies included mixed PD samples, while four focused specifically on patients with BPD. Most studies included patients with BPD and a comorbid diagnosis of PTSD (De Jongh and Slotema, 2026). We will discuss these studies in the context of treatment goals below.

**Table 1:** Clinical studies investigating the effectiveness of EMDR in patients with a personality disorder.

Study (design)	*N* (Total)	% PD at baseline	PD assessment	Treatment(s)	Study duration	No. of sessions	Session length (min)	Main outcome
Hafkemeijer et al. (2020), RCT	97 (EMDR *n* = 51; WL *n* = 46)	PD (23.7% BPD)	SCID-5; PTSD excluded via MINI	Brief EMDR therapy vs. waitlist, followed by TAU	5-week treatment period + 3-month follow-up	5	90	Greater improvements vs. waitlist; effects maintained after TAU; dropout 2%
Hofman et al. (2025) and Hafkemeijer et al. (2025), RCT	159	PD (BPD 32.9%)	SCID-5-PD	EMDR vs. waiting list	3 months follow-up	10 sessions during 5 weeks	90	Greater PTSD/PD symptom reduction; dropout 5%; BPD remission 44%
Snoek et al. (2025), RCT	124	≥4 BPD symptoms + PTSD	Structured assessment	EMDR vs. EMDR+DBT	12 months	12–18; +DBT intensive	75	Comparable outcomes; lower dropout EMDR-only (25% vs. 61%)
Nvo-Fernandez et al. (2025), RCT	76	BPD symptoms + PTSD	Not reported	EMDR vs. CBT (teletherapy)	Post-treatment	Not specified	Not reported	Both improved symptoms; dropout EMDR 76% vs. CBT 64%
Slotema et al. (2019)	47	22 BPD, 25 other PD	DSM-IV-TR	EMDR + TAU	15 weeks	1–15 (median 4)	60–90	Improvement PDS, DES, ISI; dropout 32%
De Jongh et al. (2020)	35	BPD symptoms + PTSD	BSL-23	PE, EMDR, psychoeducation	8 days	8 sessions	Varied	CAPS & BSL-23 decrease; no dropout
Kolthof et al. (2022)	45	BPD + PTSD	SCID-5-P	PE, EMDR, psychoeducation	1 year	8 sessions	Varied	Improvement CAPS-5, BPDSI-IV; remission 71%
Wilhelmus et al. (2023)	12	BPD + PTSD	SCID-II	EMDR + psychotherapy	15 weeks	8 + 15 sessions	90	Improvement PCL-5, BSCL, SDS; dropout 17%
Gielkens et al. (2024)	15	Elderly PD + PTSD	SCID-II	EMDR ± TAU	9 months	18 sessions	60	CAPS-5 decrease; PD remission 31%

### Effects of EMDR therapy on comorbid PTSD symptoms

The results of the first (uncontrolled) study that examined the pre- to post-treatment effects of EMDR therapy as an add-on to treatment as usual for PDs showed that the addition of EMDR to treatment-as-usual in patients with PDs and comorbid PTSD was associated with significant reductions in PTSD severity, dissociation, and insomnia, with large effect sizes for PTSD and insomnia (Slotema et al., 2019). The number of sessions was determined jointly by therapist and patient, with a median of only four sessions per person. Of particular interest was the observed improvement in the severity of auditory verbal hallucinations in a subgroup of participants. Furthermore, contrary to common concerns, patients with dissociative symptoms benefited safely from EMDR therapy, in that levels of dissociation decreased, and that no hospitalizations due to severe complications were reported. Comparable reductions in PTSD symptoms were found when EMDR therapy was added to individual psychotherapy for BPD immediately after admission to a specialized PD treatment department (Wilhelmus et al., 2023). Likewise, positive results were reported in an uncontrolled study among older adults with PDs (Gielkens et al., 2024). In addition, EMDR therapy, combined with prolonged exposure, physical exercise, and psychoeducation as part of a short-term intensive inpatient trauma-focused treatment program of eight treatment days, yielded strong reductions in PTSD symptoms (De Jongh et al., 2020; Kolthof et al., 2022). Remission rates for PTSD in these studies ranged from 11 to 73% (Snoek et al., 2025; Kolthof et al., 2022; Hafkemeijer et al., 2025; Slotema et al., 2019).

### Effects of EMDR therapy on personality disorder symptoms

Drawing on the AIP model, it is logical to assume that EMDR therapy is not only beneficial for patients with PD and comorbid PTSD. The results of six studies support this notion. One RCT included only patients with a PD without PTSD (Hafkemeijer et al., 2020). In this randomized controlled trial, brief EMDR therapy (5 sessions; 7.5 h total) applied immediately after admission, produced significant and clinically meaningful improvements in patients with a PD without PTSD, compared to a waiting-list control condition. Medium within-group effect sizes were observed post-treatment for psychological symptoms, psychological distress, and personality dysfunction, whereas changes in the control group were minimal or absent. Between-group analyses showed consistent advantages for EMDR therapy, with moderate effect sizes at post-treatment and sustained differences at 3-month follow-up after treatment-as-usual, despite the very brief EMDR dosage. A subsequent study among patients with and without PTSD showed similar results (Hofman et al., 2025). This multicenter RCT offered 10 standalone EMDR sessions delivered over 5 weeks. The results showed that EMDR therapy was superior to a waiting-list control in reducing PD symptom severity, with consistent improvements observed post-treatment and at 3-month follow-up across clinician-rated and self-reported measures. Effect sizes were small-to-moderate between groups but increased to moderate-to-large within the EMDR group over time, indicating sustained and clinically meaningful change. In addition to the results for the whole group, positive findings could be revealed in subgroups with avoidant PD and patients diagnosed with ‘other specified PD’. Improvements in personality functioning were also reported in older adults with PDs following EMDR therapy (Gielkens et al., 2024). The effects of EMDR therapy on specific PD categories have been investigated in a number of studies, most of which included patients with BPD (De Jongh and Slotema, 2026). Meaningful reductions in BPD symptoms were observed across all of these studies (Hofman et al., 2025; Snoek et al., 2025; Nvo-Fernandez et al., 2025; De Jongh et al., 2020; Kolthof et al., 2022), whereas remission of BPD diagnoses ranged from 57 to 73% (Kolthof et al., 2022; Hofman et al., 2025). Remission of mixed PD sample was 44% for adults and 31% for older adults (Gielkens et al., 2024). In a subgroup of patients with avoidant PD, 35% no longer met diagnostic criteria after treatment (Hofman et al., 2025).

### Evaluating the added value of concurrent psychotherapy

An RCT conducted by Snoek et al. (2025) is particularly informative because it is the only study that examined the additional value of concurrent DBT in patients with BPD and comorbid PTSD. In this randomized controlled trial both standalone EMDR and concurrent EMDR+DBT produced large and clinically significant reductions in PTSD and BPD symptoms, alongside substantial improvements in quality of life, sustained up to one-year follow-up. No significant differences were observed between conditions, indicating that the addition of DBT did not enhance outcomes beyond EMDR alone. Both interventions yielded comparable improvements across secondary outcomes, although global functioning improved more consistently in the EMDR-only condition. No significant differences emerged between EMDR therapy alone and EMDR therapy combined with DBT. This is a notable finding given the large difference in treatment intensity: 12–18 EMDR sessions versus 12–18 EMDR sessions plus 48 DBT group sessions and 29 individual sessions over the course of 1 year. Overall, the findings indicate that brief, standalone EMDR is a safe, efficient, and sufficiently potent intervention for patients with PTSD and comorbid BPD symptoms, with no added benefit, and reduced acceptability, from concurrent DBT.

### Dropout from EMDR therapy

Dropout rates of studies investigating the effectiveness of EMDR therapy for patients with a PD varied between 0 and 32%, with a weighted mean dropout rate of approximately 18%. The lowest dropout rates (5% or less) were found in studies with the shortest treatment duration, namely 5 weeks or less (Hafkemeijer et al., 2020, 2025; De Jongh et al., 2020; Kolthof et al., 2022). Notably, dropout rates were substantially higher (61%) in a study in which EMDR therapy was combined with DBT (Snoek et al., 2025), suggesting reduced feasibility of the more intensive concurrent treatment. A striking exception in the literature on the trauma-focused treatment of PDs is the very high dropout rate of 76% reported by Nvo-Fernandez et al. (2025). Methodological limitations substantially constrain the interpretation of this finding as BPD was not established through structured clinical interviews; but a self-report questionnaire. Furthermore, the study lacked an explicit description of EMDR case conceptualisation and target memory selection, an omission that is particularly consequential in the context of treating complex personality pathology with trauma-focused interventions.

### How safe is EMDR therapy?

Clinicians are often hesitant to offer trauma-focused therapy to patients with PDs because of concerns that symptoms or maladaptive behaviors, such as self-harm and suicidal behavior, may worsen. Current evidence does not support these concerns. A meta-analysis by Slotema et al. (2020) found no increase in self-injurious behavior or hospital admissions during trauma-focused treatment in this population. Similarly, there is no evidence that symptom exacerbations occurs more frequently when EMDR therapy is applied. To the contrary, although session-to-session symptom exacerbations in psychological distress and suicidal ideation does occur, these are less frequent and less severe when EMDR is applied compared to a waiting list control condition (Hafkemeijer et al., 2024). In the latter group deterioration was found to be more common (e.g., any exacerbation: 10% vs. 28% for distress; 28% vs. 43.5% for suicidality). Likewise, post-treatment worsening was rare in the EMDR group (≈2%), and continuation of therapy following temporary exacerbations was typically associated with subsequent symptom reduction (Hafkemeijer et al., 2024). Hence, these findings provide strong evidence that EMDR therapy is a safe intervention for patients with PDs and does not increase the risk of symptom exacerbation, challenging prevailing clinical concerns about trauma-focused treatment in this population.

## Discussion

One aim of this paper was to present the rationale for an alternative perspective on the development, persistence, and treatment of patients with a personality disorder (PD). This is grounded in a scientific base indicating that adverse childhood experiences, including emotional abuse and neglect, are not only highly prevalent in this population, but are also associated with an increased likelihood of developing a PD. In this regard, Shapiro’s Adaptive Information Processing (AIP) model offers a coherent explanatory framework as it poses that stressful experiences may remain insufficiently processed and become organized into maladaptive memory networks that increasingly dominate perception, emotion, and behavior (Shapiro, 2001; De Jongh et al., 2024a). Especially in interactions with significant others, these networks can be activated, evoking intense affective states and maladaptive responses to present-day situations. Such responses may damage relationships and generate new stressors, which in turn reinforce these maladaptive networks. In this way, PD symptoms may arise, intensify, and persist over time (De Jongh et al., 2024a).

Because, from this perspective, eye movement desensitization and reprocessing (EMDR) therapy may reduce PD symptoms by targeting the maladaptively stored memories underlying them, a second aim of this paper was to provide an overview of the available literature on the effects of EMDR therapy in this population. The studies reviewed here show that EMDR therapy as a stand-alone intervention, initiated shortly after admission to specialized PD treatment settings, resulted in significant reductions in PD symptoms within a relatively short time frame and with very low dropout rates (5%). Furthermore, the results showed that in response to treatment a substantial proportion of patients no longer met diagnostic criteria for PD, with remission rates up to 70% at one-year follow-up in some studies. Moreover, EMDR therapy appeared safe with no adverse events. Although research shows that temporary symptom increase and suicidal ideation can occur, studies show that this is more likely to happen if people do not undergo therapy (Hafkemeijer et al., 2024).

Despite these promising findings, several limitations warrant consideration. First, although the current evidence base is encouraging, there remains a clear need for larger-scale randomized controlled trials (RCTs) in patients with PDs, both with and without comorbid post-traumatic stress disorder (PTSD), preferably including long-term follow-up assessments. Second, most studies have been conducted in highly specialized treatment settings with substantial expertise in trauma-focused interventions, which may limit generalizability to routine clinical practice. Finally, studies investigating EMDR therapy in PD populations without PTSD have predominantly relied on waitlist control conditions. As no studies have directly compared EMDR therapy with psychotherapies specifically developed for PDs, future research should prioritize trials with active control conditions. Given evidence that EMDR may yield clinical benefits more rapidly than some trauma-focused treatments (Nijdam et al., 2012) and is among the most cost-effective interventions in this domain (Mavranezouli et al., 2020; Simpson et al., 2025), it is critical to examine whether EMDR can achieve outcomes comparable to PD-specific psychotherapies while requiring less treatment time and potentially reducing dropout rates. Clearly, these considerations are particularly relevant in light of the growing pressure on mental health care systems, where limited treatment capacity increasingly necessitates shorter and less intensive interventions. This shift is driven not only by practical constraints, such as waiting lists and workforce shortages, but also by an expanding empirical base supporting brief, trauma-focused approaches. However, it is equally important to acknowledge that short-term or lower-intensity interventions will not be sufficient for all individuals with a PD. For a subset of patients who require more intensive or longer-term treatment to achieve meaningful and sustained improvement, more extensive treatment pathways, whether or not they include trauma-focused psychotherapy, should remain available, particularly when earlier interventions have proven insufficient.

## Conclusion

The present findings provide compelling evidence that EMDR therapy is an effective, safe, and efficient intervention for individuals with PDs. By targeting maladaptively stored traumatic memories, EMDR therapy appears to address core etiological processes underlying PD pathology, resulting in broad improvements in psychological functioning. These findings challenge traditional assumptions about the treatment of PDs and support the integration of trauma-focused approaches into standard care. Future research should focus on larger, well-controlled trials, longer follow-up periods, and the identification of moderators and mediators of treatment response to further refine and optimize this promising treatment approach.
